# Structure of Merkel Cell Polyomavirus Capsid and Interaction with Its Glycosaminoglycan Attachment Receptor

**DOI:** 10.1128/JVI.01664-19

**Published:** 2020-09-29

**Authors:** Niklas J. Bayer, Dovile Januliene, Georg Zocher, Thilo Stehle, Arne Moeller, Bärbel S. Blaum

**Affiliations:** a Interfaculty Institute of Biochemistry, University of Tübingen, Tübingen, Germany; b Department of Structural Biology, Max Planck Institute of Biophysics, Frankfurt am Main, Germany; c Vanderbilt University School of Medicine, Nashville, Tennessee, USA; International Centre for Genetic Engineering and Biotechnology

**Keywords:** MCPyV, X-ray crystallography, electron microscopy, glycosaminoglycans, polyomavirus, virus-host interactions

## Abstract

Merkel cell polyomavirus (MCPyV) is a human double-stranded DNA tumor virus. MCPyV cell entry is unique among members of the polyomavirus family as it requires the engagement of two types of glycans, sialylated oligosaccharides and sulfated glycosaminoglycans (GAGs). Here, we present crystallographic and cryo-electron microscopic structures of the icosahedral MCPyV capsid and analysis of its glycan interactions via nuclear magnetic resonance (NMR) spectroscopy. While sialic acid binding is specific for α2-3-linked sialic acid and mediated by the exposed apical loops of the major capsid protein VP1, a broad range of GAG oligosaccharides bind to recessed regions between VP1 capsomers. Individual VP1 capsomers are tethered to one another by an extensive disulfide network that differs in architecture from previously described interactions for other PyVs. An unusual C-terminal extension in MCPyV VP1 projects from the recessed capsid regions. Mutagenesis experiments show that this extension is dispensable for receptor interactions.

**IMPORTANCE** The MCPyV genome was found to be clonally integrated in 80% of cases of Merkel cell carcinoma (MCC), a rare but aggressive form of human skin cancer, strongly suggesting that this virus is tumorigenic. In the metastasizing state, the course of the disease is often fatal, especially in immunocompromised individuals, as reflected by the high mortality rate of 33 to 46% and the low 5-year survival rate (<45%). The high seroprevalence of about 60% makes MCPyV a serious health care burden and illustrates the need for targeted treatments. In this study, we present the first high-resolution structural data for this human tumor virus and demonstrate that the full capsid is required for the essential interaction with its GAG receptor(s). Together, these data can be used as a basis for future strategies in drug development.

## Abstract (precis)

The MCPyV genome was found to be clonally integrated in 80% of cases of Merkel cell carcinoma (MCC), a rare but aggressive form of human skin cancer, strongly suggesting that this virus is tumorigenic. In the metastasizing state, the course of the disease is often fatal, especially in immunocompromised individuals, as reflected by the high mortality rate of 33 to 46% and the low 5-year survival rate (<45%). The high seroprevalence of about 60% makes MCPyV a serious health care burden and illustrates the need for targeted treatments. In this study, we present the first high-resolution structural data for this human tumor virus and demonstrate that the full capsid is required for the essential interaction with its GAG receptor(s). Together, these data can be used as a basis for future strategies in drug development.

## INTRODUCTION

Merkel cell polyomavirus (MCPyV) is a member of the *Polyomaviridae*, a large family of small double-stranded DNA viruses that infect mammals, birds, and fish. Currently, 12 polyomaviruses (PyVs) are known to asymptomatically infect humans. Occasionally, they can cause devastating diseases such as polyomavirus-associated nephropathy (BK polyomavirus [BKPyV]) or progressive multifocal leukoencephalopathy (JC polyomavirus [JCPyV]) in immunosuppressed patients. The high seroprevalence of about 80% for some viruses and the fact that *Polyomaviridae* are a rapidly expanding family (1, 2) make PyVs a considerable hurdle for the growing number of immunotherapies (3, 4).

The MCPyV genome was discovered in 2008 in Merkel cell carcinoma (MCC), a rare but aggressive skin cancer. It is clonally integrated into the genomes of about 80% of MCCs (5, 6) and was suggested to play a role in tumorigenesis. The MCPyV tropism and the cellular origin of MCPyV-positive MCCs are still under debate, but contrary to its name, the virus does not infect Merkel cells, skin-embedded mechanoreceptors (7–11).

Most PyVs utilize sialic acid-capped components of the plasma membrane as primary receptors for attachment and/or entry (12–16). Often, gangliosides act as PyV receptors (for instance, for simian virus 40 [SV40] [14], murine PyV [mPyV] [14], and BKPyV [13]), but glycoproteins can also fulfill this role (JCPyV) (12). On the virus side, PyV-receptor interactions are mediated entirely by the major capsid protein VP1, which forms the capsid outer layer. Sialic acid binding sites are typically composed of extended VP1 loops that form the most exposed part of the capsid (15, 17), and this is also the case for MCPyV, which binds specifically to glycans containing α2-3-linked sialic acid (18). However, the initial attachment of MCPyV is mediated not by sialic acid but primarily by sulfated glycosaminoglycans (GAGs) such as heparan sulfate (HS) and chondroitin sulfate (CS) (16). Noncomplex gangliosides like GM3 are nevertheless also required for MCPyV transduction of cultured cells, marking MCPyV as a virus with an unusual requirement for two distinct glycan receptors (16, 18, 19) as well as the only PyV with a strict requirement for GAGs.

The individual sialic acid-VP1 interaction is typically weak (in the millimolar *K_d_* [dissociation constant] range [20]) but compensated for by the high multivalency of the icosahedral capsid (360 VP1 copies). VP1 assembles into 72 pentameric capsomers, giving rise to small capsids of 45 to 50 nm in diameter. Varying numbers of the minor capsid protein VP2 and its N-terminally truncated variant VP3 associate with the interior of the capsomers, with a maximum ratio of VP1 to VP2/VP3 molecules of 5:1. VP3 is not produced by MCPyV-infected cells, possibly due to an unusually weak Kozak sequence (21). On the other hand, the MCPyV VP1 sequence harbors a 37-amino-acid (aa) C-terminal extension with unknown function and no detectable homology.

In order to investigate these unique features of MCPyV, we produced highly homogeneous virus-like particles (VLPs), which enabled us to solve crystallographic as well as cryo-electron microscopy (cryo-EM) structures and to analyze its unusual receptor usage. The structural analyses reveal a unique and extensive disulfide pattern in the MCPyV capsid that indicates a close evolutionary relationship to mPyV rather than SV40, contrary to BKPyV and JCPyV. Ligand-based nuclear magnetic resonance (NMR) experiments suggest promiscuous GAG binding to the recessions of the MCPyV capsid, similar to GAG binding by human papillomavirus 16 (HPV-16). Mutagenesis experiments show that the unique MCPyV VP1 C-terminal extension, which projects from the intercapsomer recessions, does not participate in receptor binding.

## RESULTS

### Production of highly homogeneous VLPs for atomic structure determination.

**FIG 1:**
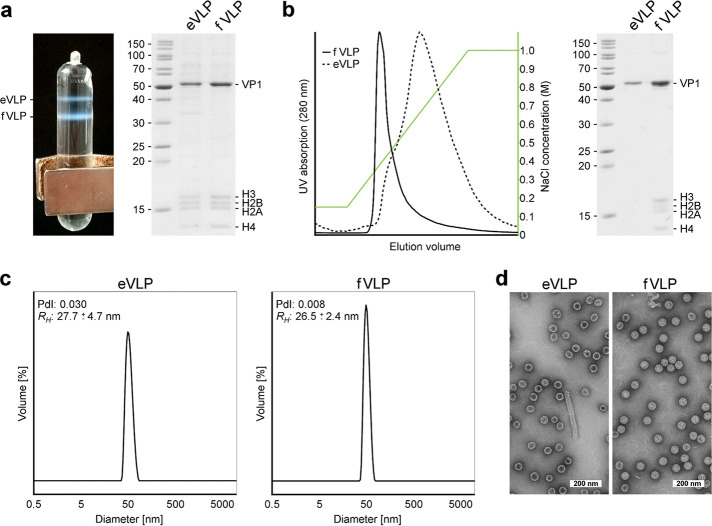
Isolation and biochemical characterization of MCPyV VLPs. (a) Separation of empty and filled virus-like particles by isopycnic density gradient centrifugation. *In vitro* assembly and maturation of VLPs were derived from 293 TT cells overexpressing the full-length MCPyV major capsid protein VP1 and initially isolated by discontinuous density gradient centrifugation (data not shown). (b) Removal of histones from eVLPs by monolithic cation exchange chromatography. Normalized UV absorptions of fVLPs and eVLPs are represented as indicated. (c) Size distribution of purified VLPs derived from dynamic light scattering. The polydispersity index (PdI) of the samples and the calculated hydrodynamic radius (*R_H_*) are indicated. (d) Negative-stain transmission electron micrographs of purified VLPs.

Established protocols for the production of PyV and papillomavirus VLPs in 293 TT cells (22, 23) were successfully modified in order to obtain structural-biology-quality MCPyV VLP samples. Two species of VLPs differing in weight could be separated by isopycnic density gradient centrifugation and were characterized biophysically. Particles of higher densities were revealed to be filled with histone-DNA complexes (filled VLPs [fVLPs]), unlike the lower-density particle fraction (empty VLPs [eVLPs]) (Fig. 1a and b). Unspecific histone-DNA complexes copurified along with MCPyV VLPs were removed by an additional monolithic ion exchange chromatography step (Fig. 1b). Here, fVLPs eluted at low ionic strength in one sharp peak and proved highly homogeneous in negative-stain transmission electron microscopy (TEM) and dynamic light scattering (DLS) experiments. Empty particles, in contrast, eluted at a higher and much broader ionic strength range and appeared less homogeneous. Occasionally, we observed copurification of elongated, tubule-like structures composed of VP1 capsomers along with eVLPs (Fig. 1d). Structurally similar tubules were observed previously in mPyV-infected cells, where they were assigned a role as “viral factories” (24, 25). For this reason and because of their generally higher homogeneity, we utilized exclusively filled particles for crystallographic and cryo-EM studies. Both empty and filled particles were, however, compared with respect to GAG binding in ligand-based NMR experiments.

### GAG binding takes place in the capsid recessions and is promiscuous.

**FIG 2:**
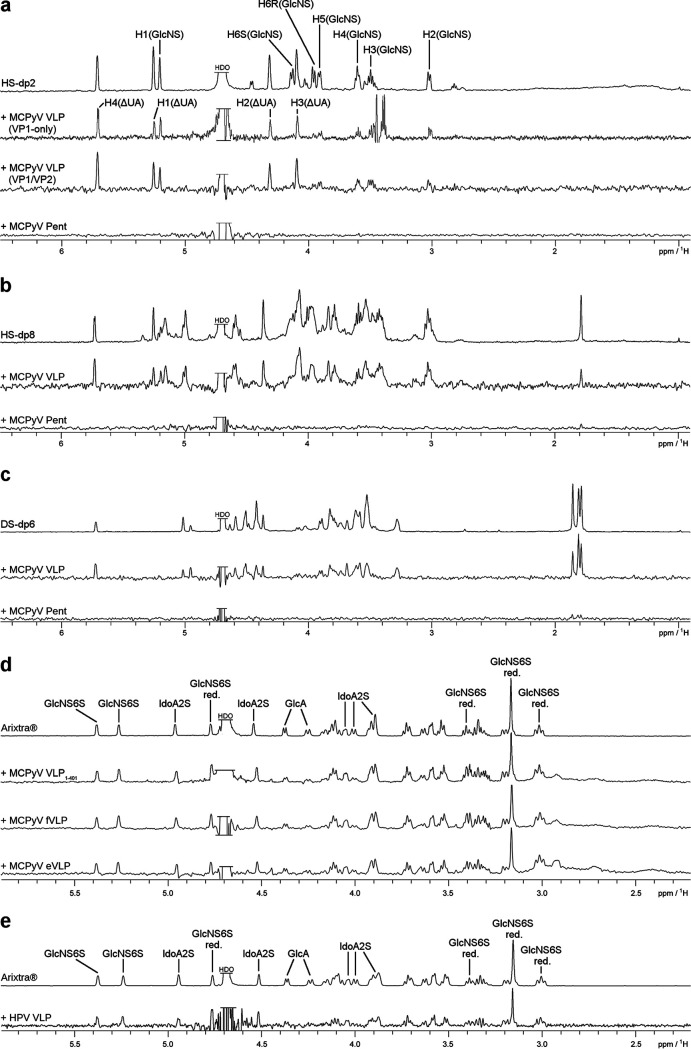
MCPyV and HPV VLP binding to GAG oligosaccharides. (a) Heparan sulfate disaccharide (HS-dp2) one-dimensional (1D) ^1^H NMR spectrum (top) and STD-NMR spectra of HS-dp2 with MCPyV VP1-only VLPs, VP1/VP2 VLPs, and MCPyV VP1 pentamers. (b) Heparan sulfate octasaccharide (HS-dp8) 1D ^1^H NMR spectrum (top) and STD-NMR spectra of HS-dp8 with MCPyV VLPs and MCPyV VP1 pentamers. (c) Dermatan sulfate hexasaccharide (HS-dp6) 1D ^1^H NMR spectrum (top) and STD-NMR spectra of DS-dp6 with MCPyV VLPs and MCPyV VP1 pentamers. (d) 1D ^1^H NMR spectrum of Arixtra in MCPyV NMR buffer (top) and STD-NMR spectra of Arixtra with the MCPyV VLP_1–401_ mutant, filled MCPyV VLPs, and empty MCPyV VLPs. (e) 1D ^1^H NMR spectrum of Arixtra in HPV-16 NMR buffer (top) and STD-NMR spectrum of Arixtra with HPV-16 VLPs. The HDO sample was truncated for better visibility. The denotation *red.* refers to the reducing-end GlcNS6S.

As previously shown crystallographically, and similar to other sialic acid-PyV VP1 complexes (17, 26), the sialic acid binding site in MCPyV is located at the outermost rim of the capsomers, formed by the VP1 apical variable loops (18). This interaction was also studied using ligand-based NMR spectroscopy (saturation transfer difference NMR [STD-NMR]), which allowed a detailed comparison of the MCPyV and BKPyV sialic acid glycan binding epitopes in solution (13). Although both viruses exhibit overlapping sialic acid glycan specificities, NMR and crystallography identified differing epitopes for the engagement of the same glycan (13, 18, 27). In particular, and nontypically for PyV-sialic acid interactions, MCPyV VP1 binds not only to the nonreducing-end sialic acid cap of human glycans (usually Neu5Acα) but also to “internal” Neu5Acα2-3Gal epitopes, for instance, in the GD3 tetrasaccharide (Neu5α2-8Neu5Acα2-3Galβ1-4Glc; underlining indicates additional binding region of MCPyV to GD3 tetrasaccharide) (18). Upon repetition of the experiment using GAG oligosaccharides rather than sialylated glycans, no interaction was observed, indicating that isolated VP1 capsomers are unable to form a viable binding site for GAGs. In contrast, intact MCPyV capsids yielded strong STD-NMR signals for a variety of GAG oligosaccharides (Fig. 2a to c).

MCPyV VLPs bound to a heparin-derived disaccharide (dp2) yielded STD-NMR spectra that suggest a larger contribution from the unsaturated uronic acid (ΔUA) ring to the interaction than from the sulfated glucosamine (GlcNS6S) ring (Fig. 2a). We further probed the binding of MCPyV VLPs to the highly sulfated, commercially available pentasaccharide Arixtra. Here, we observed STD-NMR signals throughout the full length of the pentasaccharide, with the nonsulfated pyranose, the glucuronic acid ring (GlcA), receiving less saturation than the other four, sulfated pyranoses (Fig. 2d). No significant differences in STD-NMR experiments between empty and filled MCPyV VLPs were observed, nor did we detect differences in binding between VP1-only- and VP1/VP2-containing VLPs (Fig. 2a).

MCPyV also bound to nonhomogeneous, size-defined heparin octasaccharide (HS-dp8) and to size-defined dermatan sulfate hexasaccharide (DS-dp6) (Fig. 2b and c). The fact that VLPs, but not pentameric VP1, bind to GAGs suggests that the interaction may be mediated by the capsid recessions rather than those exposed capsid regions that contain the sialic acid binding site. Strikingly, the HPV-16 VLP Arixtra binding epitope is indistinguishable from the MCPyV VLP Arixtra epitope (Fig. 2d and e).

### Role of the VP1 C-terminal extension.

**FIG 3:**
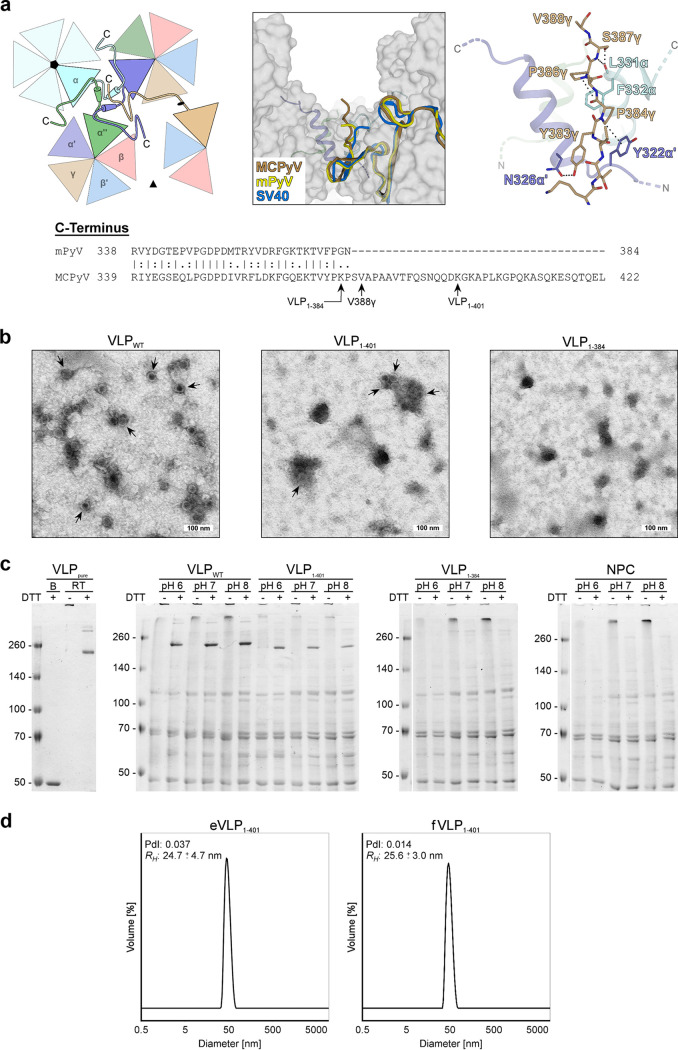
The VP1 C terminus is essential for MCPyV VP1 integrity. (a) Sequence alignment of the MCPyV and mPyV VP1 C termini and conformation of the MCPyV C terminus exemplified by the γ-monomer. The C termini of MCPyV, mPyV, and SV40 γ-monomers are located at the three-helix bundle of the α-α′-α″ interface around the icosahedral 5-fold axis (left schematic representation) and point to the recessed regions between capsomers (middle). The conformations are similar, but the structure of MCPyV lacks 35 C-terminal amino acids (388 aa of 423 aa) after the last visible stretch shown here, in contrast to mPyV (362 aa of 362 aa) and SV40 (383 aa of 384 aa). The last visible residues (Lys380γ-Val388γ) of MCPyV are stabilized by hydrogen bonds and CH-π interactions (right). Stop codon positions of the MCPyV VLP mutants and the last visible residue (Val388γ) of the MCPyV VLP crystal structure are indicated. (b) Negative-stain transmission electron micrographs of C-terminally shortened mutants of MCPyV VLPs (VLP_1–401_ and VLP_1–384_) in comparison with the wild type (VLP_WT_). Shown are cell lysates after the initial high-salt precipitation step. Arrows indicate capsid assembly. (c) Comparison of MCPyV wild-type (VLP_WT_) and truncated (VLP_1–401_ and VLP_1–384_) VP1 isolates at different pHs via nonreducing SDS-PAGE. DTT treatment of the lysates at room temperature leads to a partial decay of VLPs to pentamers (∼250 kDa). In contrast to completely denatured MCPyV monomers (B) (lane 1), capsids of untreated samples remain intact and do not enter the gel. Purified VLPs (VLP_pure_) and lysates of untransfected 293 TT cells (NPC) were used as positive and negative controls. (d) Size distribution of purified VLP_1–401_ derived from dynamic light scattering. The polydispersity index (PdI) of the samples and the calculated hydrodynamic radius (*R_H_*) are indicated.

Compared to mPyV, SV40, JCPyV, and BKPyV, the MCPyV VP1 sequence harbors a 37-amino-acid C-terminal extension with unknown function (Fig. 3a) and devoid of homology, as judged by BLAST (https://blast.ncbi.nlm.nih.gov) searches of protein sequence databases.

Neither in the crystallographic electron density nor in the cryo-EM map was this extension visible. Instead, the most-C-terminal residue resolved in either map is Val388γ (from the so-called γ-chain [see below for an explanation of the chain annotation]), meaning that a stretch of 35 amino acids is completely invisible and thus most likely flexible. Up to this amino acid, the C-terminal structure of MCPyV VP1 resembles those of SV40, mPyV, and BKPyV VP1s. However, it is clear from the electron density and electron potential maps that the missing extension must be located on the exterior of the capsid (Fig. 3a). Hence, it is potentially available for interactions with host factors. In order to probe the function of the strikingly basic C terminus (theoretical pI 9.4), we generated a VP1 mutant terminating at residue 384 (VLP_1–384_) for STD-NMR analysis. To our surprise, however, this truncated VP1 protein does not assemble into stable VLPs and is, in fact, not expressed at all (or is expressed at very low levels) (Fig. 3b and c). Therefore, we designed another mutant with only the second, more basic stretch missing (VLP_1–401_). This VP1 truncation did not impact VP1 stability, i.e., capsid integrity, with VLP preparations yielding slightly smaller amounts but homogeneity similar to that of the wild-type (WT) protein (Fig. 3b to d). STD-NMR experiments with the VLP_1–401_ mutant revealed, however, that GAG binding was also intact despite the lack of the 22 most-C-terminal amino acids (Fig. 2d). We therefore conclude that the unusually long VP1 C terminus is not solely responsible for initial receptor binding but that its deletion affects the stability of VP1.

### Structure of MCPyV capsid reveals extensive disulfide bridging.

**FIG 4:**
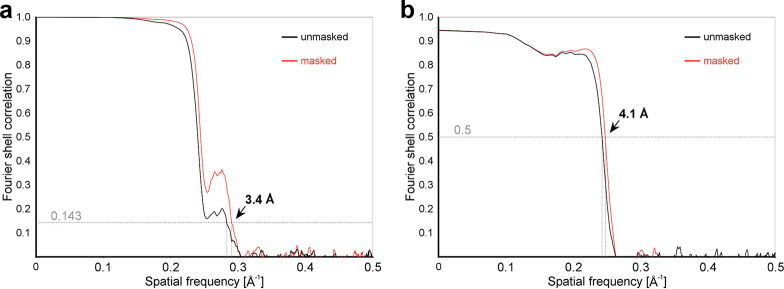
Cryo-EM data and model quality analysis. (a) Fourier shell correlation (FSC) curves between independently refined reconstructions of MCPyV VLPs. The overall resolution was estimated via a criterion of an FSC of 0.143 to be 3.4 Å. (b) FSC curves calculated between the refined model and the full map. Fourier coefficients display only minor differences up to 4.1 Å (FSC = 0.5).

**FIG 5:**
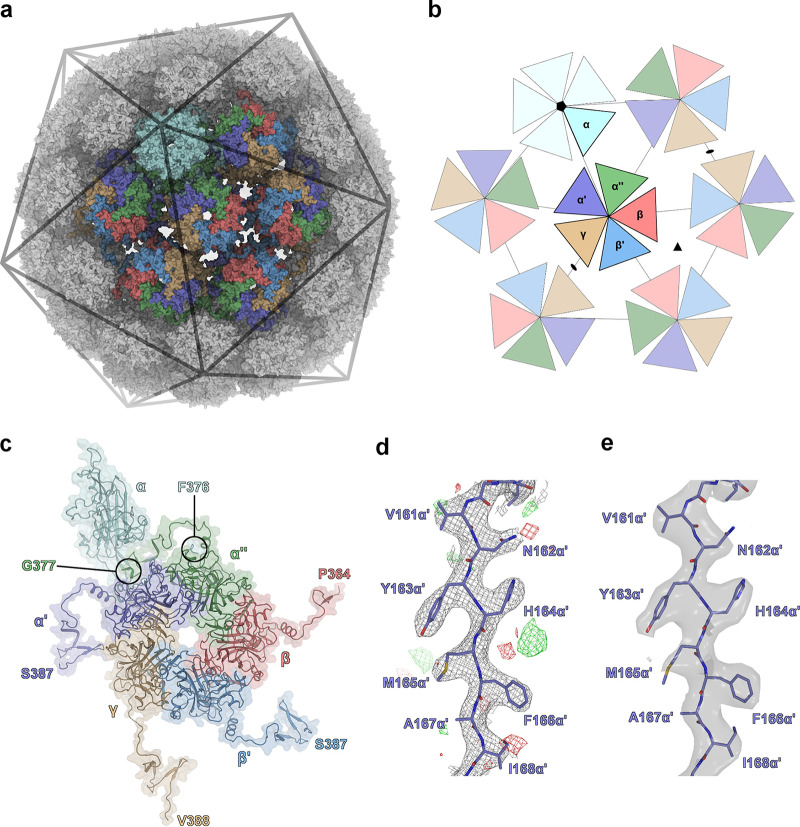
Assembly, crystal, and cryo-EM structures of the MCPyV capsid. (a) Crystal structure of the MCPyV capsid consisting of 360 VP1 copies shown in a surface representation. Owing to icosahedral T=7d symmetry, 6 different monomer conformations can be distinguished within the 72 pentameric capsomers: α (cyan), α′ (dark blue), α″ (green), β (red), β′ (light blue), and γ (brown). The VP1 α-pentamers are located at the vertices of the icosahedron and are related by strict 5-fold symmetry. (b) Schematic representation of symmetry relations within the virus capsid. The different monomer conformations are illustrated as triangles and color-coded as described above for panel a. The 5-fold symmetry axis is indicated (⬟), along with additional icosahedral symmetry axes that are present at the global β/β′-β/β′ and γ-γ interfaces (illustrated as ▲ and ⬟, respectively). (c) Icosahedral asymmetric unit used in structure refinements. The capsid can be reconstructed by the application of icosahedral symmetry operations (60-fold). Each of the six VP1 monomers interacts with neighboring pentamers via the structurally distinct C-terminal arms, which project away from the pentamer core. The last crystallographically resolved residue of each distinct monomer is marked. (d) Representative electron density of the MCPyV VLP crystal structure at a 3.5-Å resolution. 2mF_o_-DF_c_ and mF_o_-DF_c_ maps are displayed around residues Val161α′-Ile168α′ at 1 σ and 3 σ, respectively. (e) Representative electrostatic potential map of the MCPyV VLP cryo-EM structure at a 3.4-Å resolution. The map is displayed around residues Val161α′-Ile168α′ at a contour level of 6.96 (range, −32.2 to 43.8) and shows features similar to those of the crystallographic electron density map.

Although the overall structures of all PyVs solved to date are very similar in terms of the VP1 three-dimensional fold and capsomer connectivity via the VP1 termini, the SV40 and mPyV capsids differ strikingly in their disulfide patterns. Intracapsomer disulfide bridges are observed for mPyV, while intercapsomer disulfides are present only in SV40 (28). Using the fVLP preparation, we solved crystallographic and cryo-EM structures of the outer (VP1-only) MCPyV capsid. The structures have 3.5-Å and 3.4-Å resolutions, with no significant differences between them (Tables 1 and 2, Fig. 4, and Fig. 5d and e). In PyV capsids, 12 so-called “strict pentamers,” located at the icosahedral vertices defined by the 5-fold symmetry axes, are surrounded by a total of 60 “local capsomers” in a pseudo-6-fold environment (28) (Fig. 5a and b). Local capsomers differ in their interactions, the fold adopted by the individual VP1 N and C termini, as well as the C and D beta-strand-connecting loop (CD-loop) (Fig. 5c). Overall, six VP1 conformations can be distinguished within the capsid, five from a local capsomer and one from a neighboring strict capsomer, previously annotated with the Greek letters α′, α″, β, β′, γ, and α, respectively (Fig. 5a to c) (28–30). This asymmetric unit of six VP1 chains is sufficient to reconstruct the whole capsid by applying 60-fold icosahedral symmetry. The VP1 C termini project diametrically from the capsomers’ cores and interact tightly with neighboring capsomers (the so-called “invading arm”) (28, 31).

**TABLE 1:** Crystallographic data collection and refinement^*a*^

Parameter	Description or value
Data collection statistics	
X-ray source	Diamond Light Source
X-ray detector	Pilatus 6 M
Wavelength (Å)	0.98
Space group	I 2 2 2
Unit cell axes (Å)	
*a*	554.8
*b*	561.0
*c*	573.7
Unit cell angles (α = β = γ) (°)	90
Resolution (Å)	50–3.52 (3.6–3.52)
No. of unique reflections	1,088,665 (78,618)
Redundancy	13.5 (12.6)
Completeness (%)	99.8 (98.3)
*I*/σ(*I*)	9.15 (1.06)
*R*_meas_ (%)	30.9 (288.3)
CC_1/2_ (%)	99.6 (33.2)
Wilson B (Å^2^)	97.6

Refinement statistics	
Resolution included (Å)	49.97–3.52 (3.6–3.52)
Software (version)	PHENIX (1.13)
No. of atoms	244,534
*R*_work_ (%)	23.5
Bond RMSD (Å)	0.01
Angle RMSD (°)	0.84
Ramachandran plot (%) (favored/allowed/outliers)	97.08/2.92/0.00
Rotamer outliers (%)	0.31
All-atom clash score	10.76
Avg B-factor (Å^2^)	
Protein	113.1
Ions	116.1

a Values in parentheses refer to the highest-resolution shell. RMSD, root mean square deviation.

**TABLE 2:** Cryo-EM data collection and refinement

Parameter	Value or description
Data collection	
Magnification	×75,000
Voltage (kV)	300
Electron dose (e^−^/Å^−2^)	30
Defocus angle (μm)	−0.5 to −1.5
Pixel size (Å)	1.053

Map reconstruction	
No. of particles overall	23,349
No. of particles used	22,122
Resolution (Å) (FSC = 0.143)	3.42
Map-sharpening B-factor (Å^2^)	−90

Real-space refinement	
Software (version)	PHENIX (1.14)
Model resolution (Å) (FSC = 0.143)	4.13
No. of atoms	969,900
No. of protein residues	110,701
Map-to-model CC	
CC_mask_	0.82
CC_volume_	0.82
CC_peaks_	0.74
CC_box_	0.78
Bond RMSD (Å)	0.007
Angle RMSD (°)	1.14
Ramachandran plot (%) (favored/allowed/outliers)	93.14/6.81/0.0
Rotamer outliers (%)	1.91
All-atom clash score	8.07
Avg B-factor (Å^2^)	105.92

**FIG 6:**
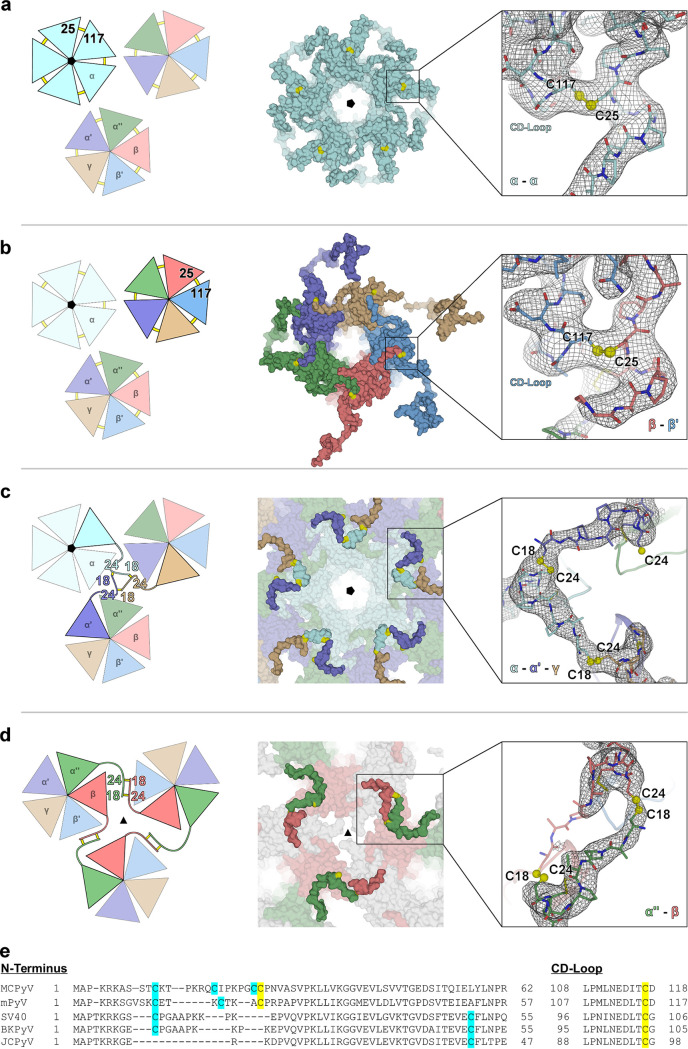
Classification of disulfide bonds formed within the MCPyV capsid: schematic, surface, and exemplary electron density representations. Electron density maps (2mF_o_-DF_c_) are adjusted to 1 σ. (a) Covalent linkages between α-monomers involve Cys25α (N terminus) and Cys117_cw_α (CD-loop) forming a circular intrapentameric disulfide bond pattern around the icosahedral 5-fold axis at the luminal surface of the virus capsid. (b) Local (nonvertex) capsomers exhibit intrapentameric connections between Cys25α′-Cys117α″, Cys25α″-Cys117β, Cys25β-Cys117β′, Cys25β′-Cys117γ, and Cys25γ-Cys117α′. (c) Interpentamer disulfide network across strict and local pentamers at the luminal surface of the virus capsid. α-, α′-, and γ-monomers of different pentamers are covalently linked via Cys18 and Cys24 in a “trinity knot”-like arrangement. Disulfide bonds between α-α′ and α-γ are well resolved in the crystal structure, while those of α′-γ are less well defined and are inferred by symmetry. (d) Interpentamer disulfide pattern between local pentamers at the luminal capsid surface of the icosahedral 3-fold axis formed by Cys18 and Cys24 of the α″- and β-monomers. The Cys18α″-Cys24β linkage is clearly visible in the electron map, while the spatial proximity of Cys18β-Cys24α″ strongly suggests the formation of a similar “disulfide handshake.” (e) Alignment of different polyomavirus sequences highlighting residues that are potentially involved in disulfide bonds. MCPyV uniquely harbors two neighboring cysteines at positions 24 and 25, thus enabling the simultaneous formation of intra- and interpentamer connections, as outlined in panels a to d. Cysteines equivalent to Cys18 in MCPyV are conserved in mPyV, SV40, and BKPyV VP1, but their VP1 proteins lack the corresponding disulfide partner in MCPyV, Cys24. Intrapentamer disulfide bridges are similar in MCPyV and mPyV, formed by cysteine residues Cys25 and Cys117 or Cys20 and Cys115, respectively. In contrast, SV40 and BKPyV pentamers are interpentamerically connected through Cys105 or Cys104, respectively, located in the CD-loop.

In MCPyV, we observed clear density for five out of potentially six disulfide bridges that connect individual VP1 chains within the strict and local pentamers, respectively, i.e., for the α-α, α′-α″, α″-β, β-β′, and γ-α′ linkages (Fig. 6a and b). For the β′-γ pair, within the local pentamers, no disulfide bridge density was observed: the β′-chain density starts only with residue 26. The arrangement and proximity of the respective chains suggest that this disulfide bridge is nevertheless formed. The MCPyV cryo-EM data further support the presence of this intrapentameric disulfide bond pattern; nonetheless, only the α-α connection is clearly visible in the electrostatic potential map. For mPyV virions, intracapsomer disulfide bridges are the only disulfides observed previously (the respective mPyV residues are Cys20 and Cys115), with no reported intercapsomer disulfides. MCPyV VP1 contains two adjacent cysteines (Cys24 and Cys25) within its N-terminal region (Fig. 6e). The crystallographic electron density shows unambiguously that only Cys25 is involved in the ringlike intracapsomer disulfide bridges, tethering each VP1 N terminus covalently to its clockwise (cw) neighbor’s CD-loop (residues 108 to 118) via Cys117, the mPyV Cys115 homolog. This observation places MCPyV in the mPyV structural clade since an equivalent disulfide pattern is observed for mPyV (28) but not for SV40 (Fig. 6e). Additionally, the MCPyV crystal structure revealed unique intercapsomer disulfide bridges, rendered possible by the presence of two additional cysteines (Cys18 and Cys24) (Fig. 6e). These two cysteines form two novel and distinct structural elements, which we term the trinity knot and disulfide handshake, respectively (Fig. 6c and d). The trinity knot is located at the interface between two local pentamers and one strict pentamer tethering each of the α-, α′-, and γ-chains to the respective other two. The disulfide handshake links two local pentamers whose chains run in an antiparallel fashion, with two simultaneous Cys18-Cys24 bridges (Cys18α″-Cys24β and Cys18β-Cys24α″). This motif is located close to the icosahedral 3-fold symmetry axis; i.e., three disulfide handshakes are close to one another. Overall, each of the 12 strict pentamers engages in 5 trinity knots (1 with each of the 5 local pentamers surrounding each strict pentamer) (Fig. 5), while each of the local pentamers forms 2 trinity knots with its respective neighboring strict pentamer and 2 with its neighboring local pentamers. Additionally, each of the 30 local pentamers that are located next to one of the 10 3-fold symmetry axes forms 2 disulfide handshakes (1 with each of the other 2 local pentamers surrounding the same 3-fold axis) (Fig. 6d). Since all other PyVs lack a residue that is equivalent to Cys24, they are most likely unable to form such an extensive disulfide pattern.

### Capsid stabilization by disulfides and Ca^2+^-chelating sites.

**FIG 7:**
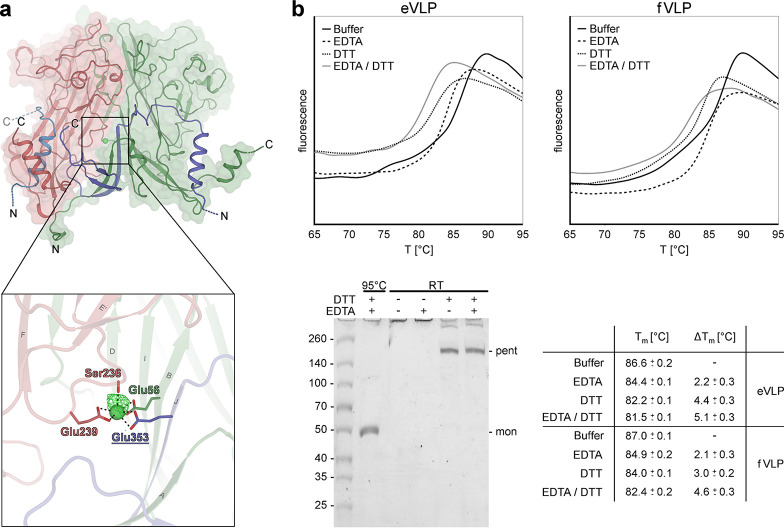
Calcium coordination and disulfide bond contribution to MCPyV capsid stability. (a) Calcium ions stabilize intrapentameric contacts via the carbonyl moiety of Ser236 and the side chain carboxyl groups of Glu239 and Glu56_ccw_. Glu353, contributed by an invading arm of a neighboring pentamer, is also involved in metal coordination, thus representing an additional interpentameric connection. The binding site is exemplified using the α″-β-monomer interface and exhibits an octahedral metal coordination geometry with Ca-O bond lengths of ∼2.63 Å, with the proximal and square plane positions exhibiting weak electron density that may reflect water molecules with incomplete occupancies (not modeled). The presence of the calcium ions was confirmed by calculating a simulated annealing mF_o_-DF_c_ omit map (green mesh) at 3 σ. (b) Contributions of calcium binding and disulfide bridges to capsid stability evaluated by thermal shift assays and SDS-PAGE. Melting curves of empty VLPs (eVLPs) and filled VLPs (fVLPs) were recorded in the presence of DTT and/or EDTA. Melting temperature (T_m_) differences were calculated in comparison to untreated samples (bottom right table). Standard deviations/precisions of the measurements are indicated. SDS-PAGE shows the partial decay of fVLPs to pentamers (pent) after disulfide bond reduction at room temperature (RT) (lanes 4 and 5). In contrast to completely denatured MCPyV monomers (mon) (lane 1), capsids of untreated (lane 2) as well as only EDTA-exposed (lane 3) samples remain intact and do not enter the gel.

Another conserved feature of PyV capsids is inter-VP1 Ca^2+^-chelating sites. A role for calcium as a promoter and stabilizer of PyV assembly was first described in *in vitro* assembly studies monitored with electron micrographs (29). No calcium ions were observed in the electron density maps of either SV40 or mPyV virion structures, possibly due to the absence of the element in the respective crystallization buffers. However, in the case of SV40, the location and chelation of two potential Ca^2+^ ions could be deduced upon crystal incubation with gadolinium ions (28). For our MCPyV capsids, we provided 1 mM calcium chloride in all buffers. The crystallographic electron density suggests the presence of only one divalent ion, coordinated by the Ser236 backbone carbonyl and Glu239 side chain carboxyl groups of one monomer as well as the Glu56 side chain of the neighboring monomer and the Glu353 side chain of an invading arm of another capsomer (counterclockwise [ccw]) [i.e., involving Ser236α′, Glu239α′, Glu56γ, and (ccw)Glu353γ] (Fig. 7a). The location of this site corresponds to SV40 calcium site 1, formed by SV40 residues Ser213, Glu216, Glu330, and Glu48/Glu46 (28).

Biochemical assays confirmed the chemical identity of the Ca^2+^ ion and the effect of bivalent cations and disulfide bridges on the capsid’s chemical and thermal stability. First, we treated MCPyV capsids with reducing (dithiothreitol [DTT]) and divalent ion-chelating (EDTA) agents and monitored structural integrity at room temperature via SDS-PAGE (Fig. 7b). While intact VLPs did not enter the gel (Fig. 7b, lane 2), we observed a distinct band at around 250 kDa upon treatment with 10 mM DTT (Fig. 6b, lane 4), possibly reflecting intact capsomers (the molecular weight [MW] of monomeric VP1 is 46.6 kDa). No effect was seen at room temperature for 50 mM EDTA (Fig. 7b, lane 3), but additional heating led to complete disassembly, yielding only monomeric VP1 (Fig. 6b, lane 1). The effect on the capsid melting temperature was significant for both reagents, as assessed by thermal shift assays (TSAs), leading to a reduction from 87.0°C for the untreated fVLPs to 84.0°C upon disulfide reduction and to 84.9°C upon incubation with EDTA (Fig. 6b). The combined effect of disulfide reduction and cation chelation further decreased the melting temperature to 82.4°C. For eVLPs, the trend was even more pronounced, with melting temperatures of 86.6°C for intact capsids, 82.2°C upon DTT and 84.4°C upon EDTA treatments, and 81.5°C for the combination of both agents.

## DISCUSSION

In this study, we aimed to further the understanding of the early steps of MCPyV infection and its unusual receptor usage of two distinct types of cell surface glycoconjugates, sialic acid-containing glycans and GAG-presenting proteoglycans. Structural information on MCPyV available to date has been obtained using assembly-incompetent VP1 pentamers (18). However, since we found GAG binding to require an intact capsid, we produced MCPyV VLPs to enable structural and functional studies of the virus-GAG interaction. These VLPs are of exceptional homogeneity, as evidenced by their ability to form well-diffracting crystals. As high-resolution structures of recombinantly produced VLPs are exceedingly rare, the production protocol established here may help facilitate structural analyses of other VLPs, thus circumventing the need for higher biosafety levels to analyze infectious virus particles.

Although both MCPyV receptors are glycoconjugates and plasma membrane constituents, they are fundamentally different biomolecules. Unlike sialylated glycoconjugates, GAGs are large and linear polysaccharides of highly but irregularly sulfated disaccharide repeats, presented by a small number of scaffold proteins (proteoglycans) (32). While binding to sialic acid by MCPyV is fairly specific (18), we observed considerable promiscuity with respect to GAG binding. One of the GAG probes employed in our STD-NMR experiments is enzymatically prepared heparin/HS-derived disaccharide (dp2 [ΔUA2Sα1-4GlcNS6S]). While dp2 represents a fully sulfated disaccharide, it differs in both pyranose ring structures in comparison to intact (high-molecular-weight) HS (through an unsaturated bond in the nonreducing ring and dynamic epimerization in the reducing-end ring). Nevertheless, an interaction between MCPyV VLPs and dp2 was unambiguously observed. MCPyV VLPs also bound to a structurally inhomogeneous heparin-derived octasaccharide preparation and DS-derived hexasaccharide as well as the synthetic, chemically pure HS-pentasaccharide Arixtra (GlcNS6Sα1-4GlcAα1-4GlcNS3S6Sα1-4IdoA2Sα1-4GlcNS6Sα-OMet, where OMet is O-coupled methyl group). For the latter compound, a more efficient saturation transfer to the four sulfated rings than to the single nonsulfated (GlcA) ring was observed. Overall, these experiments demonstrate a broad GAG binding profile for MCPyV and suggest that sulfation is an important parameter for the interaction, possibly of larger importance than the underlying GAG pyranose scaffold. We note that GAG binding to isolated, assembly-incompetent VP1 pentamers is not observable by STD-NMR, a technique capable of detecting very weak interactions (in the millimolar *K_d_* range). Furthermore, mutations in the sialic acid binding site do not affect GAG binding to correctly assembled VLPs (18, 19), also demonstrating that the GAG binding site is distant from the sialic acid binding site. Taken together, these observations suggest that the GAG binding site spans more than one VP1 pentamer and is most likely located near the capsid recession. This is in contrast to the region that engages sialic acid, which includes apical loops in an isolated VP1 pentamer. Although we think that such a scenario is unlikely, we cannot rule out the possibility that the high mutivalency of the full capsid with respect to the assembly-incompetent pentamers plays an important role in the detectability of GAG binding by MCPyV.

While we observed a direct GAG-MCPyV interaction by NMR, our crystal soaking and cryo-EM attempts to produce conclusive GAG-VLP complex structures remained unsuccessful despite the relatively high resolution obtained for the unliganded VLP structures. Recent attempts to provide HPV-16–GAG and BKPyV-GAG cryo-EM complex structures resulted in complexes with weak electron potential in the respective capsid recessions in either case, which was attributed to GAG polymers (33, 34). Instead of highly inhomogeneous polymeric GAGs, we employed the chemically homogeneous Arixtra pentasaccharide in our crystallographic and cryo-EM attempts. Therefore, and in the light of our positive NMR binding data for the same compound, we propose that the experimental difficulties that so far prevented high-resolution data on VLP-bound GAGs are not merely caused by the usage of inhomogeneous heparin preparations in the BKPyV and HPV-16 studies, as assumed by the authors of the BKPyV study (33). Instead, the fuzzy electron density could also reflect variable binding modes of chemically homogeneous and inhomogeneous GAGs within a given binding site. Strikingly, the HPV-16 VLP Arixtra binding epitope is indistinguishable from the MCPyV VLP epitope, also supporting the hypothesis that GAG binding is relatively unspecific. This scenario is in stark contrast to the highly specific interactions that restrict PyVs to a generally very small set of sialic acid glycans, as exemplified by the BKPyV/MCPyV comparison (13, 18, 27).

VP1 sequence comparison suggests that both BKPyV and JCPyV belong to the SV40 PyV clade (8), with high sequence identities (81.7% [33] and 76.7%, respectively). Structural evidence for an SV40-like disulfide pattern was recently confirmed for BKPyV (33). In contrast, MCPyV exhibits much lower similarity to either PyV prototype and more closely resembles mPyV than SV40, with VP1 sequence identities of 50% and 45%, respectively. The extensive disulfide pattern reported here exceeds the one thus far reported for other PyVs, which is not surprising given that MCPyV VP1 contains additional cysteine residues compared to other PyV VP1 sequences. However, the “disulfide ring” that interconnects individual protein chains within strict as well as local VP1 pentamers was reported for mPyV but not SV40 previously (28, 31, 35). The presence of only one Ca^2+^ binding site in the capsid is also more reminiscent of mPyV than SV40. Thus, from a structural point of view, MCPyV more closely resembles mPyV, as was suggested by sequence analysis (5).

PyV cellular trafficking was previously shown to proceed in a retrograde manner through the endoplasmic reticulum (ER) (36, 37). Presumably, disulfide bonds in the capsid are partially reduced by ER disulfide isomerases, triggering the recognition of the altered virion by the ER unfolded protein response and the translocation of the uncoating virus to the cytosol (14, 36, 38, 58, 59). Here, the low calcium concentration could promote further uncoating via destabilization of the Ca^2+^-mediated intra- and inter-VP1 interactions. Our finding that both conserved PyV capsid structural elements, disulfides and Ca^2+^-chelating sites, are important for MCPyV capsid stability suggests that this PyV-typical mechanism may also be employed during MCPyV infection. MCPyV’s extensive disulfide network may reflect an ability to withstand harsh environmental conditions, which would presumably contribute to efficient human-to-human transmission and the presence of MCPyV in the common skin microflora.

## MATERIALS AND METHODS

### Plasmids and mutant generation.

The codon-optimized VP1- and VP2-coding plasmids used were pwM (Addgene plasmid 22515) and ph2m (Addgene plasmid 22518), respectively (sequences from MCC isolate 339 [5]).

The C-terminally truncated VP1 variants VP1_1–384_ and VP1_1–402_ were produced in Escherichia coli DH5α cells by introducing additional ochre stop codons (UAA) into the pwM plasmid using site-directed mutagenesis. All plasmids were propagated in E. coli XL10-Gold cells and purified for mammalian cell transfection using the GenElute HP plasmid maxiprep kit (Sigma-Aldrich).

### Production of MCPyV VLPs.

MCPyV VLPs were purified by a modification of existing protocols (22, 23), optimized for obtaining a highly homogeneous sample suitable for structural studies. Siliconized Eppendorf cups were used throughout the purification procedure in order to minimize sample loss. Confluent 293 TT cells (22) were cultivated in Dulbecco’s modified Eagle’s medium (DMEM) (Gibco) in the presence of 10% fetal calf serum (FCS) and 250 μg/ml hygromycin at 37°C with 5% carbon dioxide saturation. Transient transfection was performed at a cell count of 4.8 × 10^7^ cells (175-cm^2^ flat-bottom flask) in serum-reduced medium (2:1 ratio of DMEM supplemented with 10% FCS and Opti-MEM [Gibco] plus 4.5 μg/ml polyethylenimine [PEI] [MW, 25,000; Polysciences Inc.]) using a 5:1 ratio of pwM and ph2m and a total amount of 89 μg DNA per culture flask. Transfection medium was exchanged to DMEM supplemented with 10% FCS after 24 h, and cells were incubated for 3 days in the absence of hygromycin. Cells were harvested by treatment with trypsin-EDTA (Gibco) and resuspended in Dulbecco's phosphate-buffered saline (DPBS) supplemented with 9.5 mM MgCl_2_. The transfection efficiency was monitored via the expression of an enhanced green fluorescent protein (eGFP) reporter gene on the pwM plasmid using fluorescence-activated cell sorting (FACS) (data not shown). Cells were lysed at a high density of >10^8^ cells/ml by resuspension in lysis buffer (DPBS supplemented with 8 mM MgCl_2_, 0.5% [vol/vol] Triton X-100, 0.2% [vol/vol] Benzonase [250 U/μl; Sigma], 25 mM ammonium sulfate [pH 9], 1 μg/ml soybean trypsin inhibitor [Sigma-Aldrich], and 1× rComplete [Roche] protease inhibitor dissolved in DPBS). VLP maturation was achieved by incubation of the lysate for 18 h at 37°C.

The crude lysate was adjusted to 850 mM NaCl and incubated for 30 min at 4°C. The soluble fraction was initially clarified for 10 min at 10,000 × *g* (4°C), filtered (0.22-μm-diameter cellulose membrane), and applied onto a discontinuous density gradient of 15% (wt/vol) sucrose–35% (wt/vol) CsCl in purification buffer (20 mM HEPES [pH 6.6], 150 mM NaCl, 1 mM CaCl_2_). VLPs were isolated from the lysate at 129,840 × *g* for 1 h (10°C) in a fixed-angle rotor (70.1 Ti rotor; Beckman).

The separation of DNA-filled VLPs (fVLPs) and empty VLPs (eVLPs) was performed via isopycnic density gradient ultracentrifugation with 4.4 M CsCl in purification buffer at 277,816 × *g* for 18 h (10°C) in a fixed-angle rotor (70.1 Ti rotor; Beckman). The resulting two VLP bands were readily visualized via visible lateral light scattering, manually extracted with a syringe, and separately dialyzed against purification buffer at 4°C. The protein concentration was estimated via the UV absorption at 260 nm (AU_260_) and 280 nm, respectively (for fVLPs, 3.84 AU_260_ = 1 mg/ml; for eVLPs, 1.09 AU_280_ = 1 mg/ml). Both species could be distinguished by their respective 260/280-nm ratios (fVLPs, ∼1.3; eVLPs, <1.0).

The VLP sample quality was further improved by performing cation exchange chromatography using a monolithic CIMacSO_3_ column (BiaSep). VLP fractions were separately loaded and eluted with a linear NaCl gradient (0.15 to 1 M NaCl, 20 mM HEPES [pH 6.6], 1 mM CaCl_2_) over 20 ml at a flow rate of 1 ml/min. The purified eVLPs and fVLPs were concentrated by ultrafiltration (100,000-MW-cutoff [MWCO] Amicon filter; Merck-Millipore) to a final concentration of 1 mg/ml, flash-frozen in liquid nitrogen, and stored at −80°C.

### Production of HPV-16 VLPs.

L1- and L2-containing HPV-16 VLPs were produced using the MCPyV VLP procedure, except for the ion chromatography step, with plasmids pshell16 and p16L1L2 (39, 40). Phosphate-buffered saline (PBS) prepared in 99% D_2_O was used as the NMR sample buffer, and VLPs were dialyzed into this buffer after the second ultracentrifugation step.

### Dynamic light scattering.

For the assessment of sample homogeneity and size estimation, concentrations of eVLPs and fVLPs were adjusted to 0.1 mg/ml (20 mM HEPES [pH 6.6], 150 mM NaCl, 1 mM CaCl_2_), and dynamic light scattering (DLS) measurements were performed on a Zetasizer Nano ZS instrument (Malvern Panalytica) using a 3-mm quartz cuvette. Each sample was measured in triplicates (15 accumulations) at 20°C in noninvasive back-scatter mode at 633 nm. The average hydrodynamic radius (*R_H_*) and the polydispersity index (PdI) were calculated based on the merged autocorrelation functions using Zetasizer software 7.11.

### Negative-stain transmission electron microscopy.

Soluble fractions of the clarified VLP lysates or purified VLP solutions at a concentration of 1 mg/ml were directly placed onto a glow-discharged EM grid (thin-bar, square, 300-mesh Cu grids with Formvar-carbon support film; Science Services). After adsorption, the grids were washed with double-distilled water and stained with 1% uranyl acetate. The grids were then examined using a Zeiss Libra 120 transmission electron microscope (Carl Zeiss, Oberkochen, Germany) operated at 120 kV.

### Thermal shift assay.

The effects of disulfide bond formation and calcium complexation on capsid stability were estimated in thermal shift assays (TSAs) performed on a QuantStudio 5 real-time PCR cycler (Applied Biosystems, Thermo Fisher Scientific). VLPs were diluted in purification buffer to a concentration of 0.1 mg/ml and incubated with 10 mM dithiothreitol (DTT) and/or 10 mM EDTA for 1 h at room temperature prior to the addition of 1× Protein Thermal Shift dye (Applied Biosystems, Thermo Fisher Scientific). The samples were equilibrated at 4°C and gradually heated to 95°C over 30 min while monitoring dye fluorescence at 586 nm. The averaged protein melting temperature for each reaction was calculated from the melting curve inflection points from three replicates using Protein Thermal Shift software v1.3 (Applied Biosystems, Thermo Fisher Scientific). All reagents used were preadjusted to pH 6.6 to avoid pH-dependent effects on the thermal stability of the VLPs.

### Nonreducing SDS-PAGE.

Nonreducing SDS-PAGE was performed using 4 to 20% Tris-glycine precast gradient gels (Rosetta Stone Biotech) or 8% Tris-glycine gels. All samples were incubated in purification or lysis buffer with 10 mM DTT and/or 50 mM EDTA in the presence of 2% (wt/vol) SDS for 1 h at ambient temperature. Per well, 0.2 μg of capsid or a 1:10 dilution of the normalized crude lysate was applied, and a heat-treated sample (5 min at 95°C) with 10 mM DTT and 50 mM EDTA was used as the VP1 monomer reference sample. The molecular weight was estimated using a Spectra multicolor broad-range protein ladder (Thermo Fisher Scientific), and electrophoresis was performed using Tris-glycine buffer (Laemmli buffer [41]) at a constant current of 35 mA/gel until the marker reached the end of the gel.

### Crystallization and data collection.

MCPyV fVLPs were crystallized in a hanging-drop vapor diffusion setup at protein concentrations of 1 to 1.5 mg/ml in a broad range of sodium citrate buffers containing 2-methyl-2,4-pentanediol (MPD) and NaCl. Diffraction was strictly crystal size dependent. The best diffraction data were obtained from a crystal grown for approximately 1 year in a solution containing 0.1 M sodium citrate (pH 4.6), 30% (vol/vol) MPD, and 0.2 M NaCl at 20°C. Crystals were flash-frozen in liquid nitrogen without an additional cryoprotectant, and X-ray diffraction data were collected at the I03 beamline of the Diamond Light Source (Didcot, UK). Diffraction images from three data sets collected on one crystal were integrated, reduced, and merged to a 3.5-Å resolution using the XDS/XSCALE program package (42).

### Phasing, model building, and refinement.

The initial phases for the MCPyV capsid structure were obtained by molecular replacement with PHASER (v2.5.0) (43) using a CHAINSAW-modified (44) search model generated from the SV40 capsid structure (Protein Data Bank [PDB] accession number 1SVA) (28). For model building, 15-fold-constraint noncrystallographic symmetry (NCS) parameterization was applied to the six conformationally unique VP1 monomers α, α′, α″, β, β′, and γ in order to reconstruct the crystal asymmetric unit of 90 VP1 chains. The model was completed by several cycles of manual real-space correction in COOT (45) (v0.8.9.1) and reciprocal space refinement using PHENIX (46) (v1.13). Each of the six monomers was separately built and copied with respect to the NCS symmetry using a Python-based PyMOL script. One rigid-body group was defined for each monomer of the asymmetric unit and used during the first macrocycle of refinement, followed by NCS constraint coordinate and group-B-factor optimization. Due to a mild deviation from a perfectly spherical shape, a different density appeared systematically in the unit cell during refinement. Therefore, the pentamer-connecting so-called invading arms of the individual VP1 chains were excluded from the NCS, and a separate NCS group was defined for the α-monomers in order to eliminate the otherwise restrained angle between the strict and local capsomers. Additionally, B-factor sharpening of −45.6 Å^2^ was applied using PHENIX.autosharp (47), which further increased the map interpretability. Model and map accuracies were evaluated by NCS-based B-factor analysis and calculation of real-space correlation coefficients (RSCCs). Figures were generated using PyMOL (v1.8.4; Schrödinger Inc.).

### Cryo-EM sample preparation, data acquisition, and processing.

Three microliters of the MCPyV sample at a concentration of ca. 10 mg ml^−1^ was applied onto freshly glow-discharged CF 2/2 grids (Microchips) and plunge-frozen in liquid ethane using Vitro bot Mark IV (Thermo Fisher). Micrographs were recorded automatically (EMU software; Thermo Fisher), using a Titan Krios microscope operated at 300 K, with a Falcon 3EC direct electron detector (Thermo Fisher) at a nominal magnification of ×75,000, corresponding to a pixel size of 1.053 Å. Dose-fractionated movies were acquired at an electron flux of 0.45 e^−^/pixel/s over 76 s (67 frames), corresponding to a total electron dose of ∼30 e^−^/Å^2^. Images were recorded in a defocus range from −0.5 to −1.5 μm. Frame-based motion correction was performed using MotionCor2 (48) with a dose filter of 0.45 e^−^/Å^2^/frame. The contrast transfer function (CTF) was estimated from the non-dose-weighted images using CTFFIND4 (49). A total of 23,349 particles were picked using Dog-picker (50) as implemented in Appion (51) and refined in *cis*TEM (52), applying icosahedral symmetry. After obtaining a consensus map, the particles were subclassified into 3 classes and locally refined. This procedure combined with CTF refinement yielded a map at a 3.4-Å resolution, containing 95% of all picked particles. The reported resolution was estimated according to the gold-standard criterion of a Fourier shell correlation (FSC) value of 0.143 (53).

### Cryo-EM model building and refinement.

The map was initially sharpened by applying a B-factor of −90 Å^2^ using a hollow-sphere mask with radii of 265 Å and −150 Å. The crystal structure of the MCPyV capsid was positioned within the cryo-EM map using rigid-body fitting in CHIMERA (54) (v.1.11.2) based on the map-to-model correlation coefficient (CC = 0.7). For model building, the map was segmented, covering the asymmetric unit, followed by a simulated annealing protocol in PHENIX (v.1.14). Several iterations of manual model correction were performed in COOT (v0.8.9.1), and the icosahedral asymmetric unit was refined using PHENIX.real_space_refine (55), applying NCS constraints. The model was evaluated by using MOLPROBITY (56), and calculations of RSCC, half-map-based FSC, model-to-map FSC (data not shown), as well as CC (Table 2) were performed using PHENIX.validation_cryoem (57). Figures were generated using PyMOL (v.1.8.4).

### NMR spectroscopy.

All STD-NMR spectra were recorded on a Bruker AVIII 600-MHz spectrometer equipped with a room-temperature probe head at temperatures of 283 K using 3-mm-internal-diameter (ID) Match tubes. The MCPyV NMR buffer contained 150 mM NaCl and 1 mM CaCl_2_ in 99% D_2_O (Cortecnet). The HPV-16 and MCPyV VP1 NMR buffer contained 150 mM NaCl and 20 mM KH_2_PO_4_ (pH 7.4) in 99% D_2_O. Samples contained about 10 μM VLPs or 20 μM VP1 and 1 mM the respective oligosaccharide(s). HS-dp2, HS-dp8, and DS-dp6 oligosaccharides were purchased from Dextra Laboratories and resuspended in 99% D_2_O to yield 40 mM stock solutions. Arixtra (Aspen Laboratories) was dialyzed in NMR buffer using 500-MWCO Slide-A-Lyzer conical cups with cellulose membranes (Thermo Fisher Scientific). Off- and on-resonance frequencies in STD-NMR experiments were set to −30 ppm and −0.5 ppm, respectively. The irradiation power and length of the selective pulse train were 57 Hz and 2 s, respectively. A strength of 3.2 kHz was employed to suppress residual protein resonances in a continuous-wave spin-lock pulse. A total of 5,000 scans were recorded, and the relaxation delay was 3 s. Prior to Fourier transformation, NMR data were multiplied with a Gaussian window function. Data were processed with TOPSPIN 3.0 (Bruker).

### Data availability.

The MCPyV structures described here were deposited in the Protein Data Bank under accession numbers 6ZLZ and 6ZML. Cryo-EM data are available at the EM Data Resource (www.emdataresource.org) under accession number EMD-11293.
